# Independent Validation of an Existing Model Enables Prediction of Hearing Loss after Childhood Bacterial Meningitis

**DOI:** 10.1371/journal.pone.0058707

**Published:** 2013-03-11

**Authors:** Rogier C. J. de Jonge, Marieke S. Sanders, Caroline B. Terwee, Martijn W. Heymans, Reinoud J. B. J. Gemke, Irene Koomen, Lodewijk Spanjaard, A. Marceline van Furth

**Affiliations:** 1 Department of Pediatric Infectious Diseases - Immunology, and Rheumatology, VU University Medical Center, Amsterdam, The Netherlands; 2 Department of Neonatology, Erasmus MC - Sophia Children’s Hospital, Rotterdam, The Netherlands; 3 Department of Medical Microbiology, Laboratory for Immunogenetics, VU University Medical Center, Amsterdam, The Netherlands; 4 Department of General Medicine, Antonius Hospital, Nieuwegein, The Netherlands; 5 Department of Epidemiology and Biostatistics and the EMGO Institute for Health and Care Research, VU University Medical Center, Amsterdam, The Netherlands; 6 Department of Pediatrics, VU University Medical Center, Amsterdam, The Netherlands; 7 Department of Pediatrics, Westfriesgasthuis, Hoorn, The Netherlands; 8 Department of Medical Microbiology, Netherlands Reference Laboratory for Bacterial Meningitis, Academic Medical Center, Amsterdam, The Netherlands; NIAID, United States of America

## Abstract

**Objective:**

This study aimed external validation of a formerly developed prediction model identifying children at risk for hearing loss after bacterial meningitis (BM). Independent risk factors included in the model are: duration of symptoms prior to admission, petechiae, cerebral spinal fluid (CSF) glucose level, *Streptococcus pneumoniae* and ataxia. Validation helps to evaluate whether the model has potential in clinical practice.

**Study design:**

116 Dutch school-age BM survivors were included in the validation cohort and screened for sensorineural hearing loss (>25 dB). Risk factors were obtained from medical records. The model was applied to the validation cohort and its performance was compared with the development cohort. Validation was performed by application of the model on the validation cohort and by assessment of discrimination and goodness of fit. Calibration was evaluated by testing deviations in intercept and slope. Multiple imputation techniques were used to deal with missing values.

**Results:**

Risk factors were distributed equally between both cohorts. Discriminative ability (Area Under the Curve, AUC) of the model was 0.84 in the development and 0.78 in the validation cohort. Hosmer-Lemeshow test for goodness of fit was not significant in the validation cohort, implying good fit concerning the similarity of expected and observed cases. There were no significant differences in calibration slope and intercept. Sensitivity and negative predicted value were high, while specificity and positive predicted value were low which is comparable with findings in the development cohort.

**Conclusions:**

Performance of the model remained good in the validation cohort. This prediction model might be used as a screening tool and can help to identify those children that need special attention and a long follow-up period or more frequent auditory testing.

## Introduction

Due to successful vaccination programs, and to spontaneous decline in *Neisseria meningitidis* serogroup B infections, the incidence of bacterial meningitis (BM) in childhood is decreasing in the Western world. Still, BM ranks among the top ten causes of death in children younger than 14 years in high-income countries. Further, developing countries account for 98% of the estimated 5.6 million disability-adjusted life years attributed to meningitis globally [1], [2]. Sensorineural hearing loss is the most common severe consequence of BM, with an incidence in children of 7–31% [3], [4], [5], [6], [7], [8], [9]. Hearing loss after BM is probably multicausal. Bacterial labyrinthitis due to dissemination of the infection from the subarachnoid space in combination with toxic or serous labyrinthitis, direct nerve fiber damage and secondary ischemic damage are thought to be part of the mechanism [10].

Especially in (young) children even mild impairment in hearing abilities may impair auditory, linguistic, communication and learning skills. Early identification of hearing loss is indispensable for effective treatment resulting in the acquisition of normal linguistic development [11], [12]. Further, cochlear ossification may complicate cochlear implantation making early diagnosis even more essential. [13], [14].

The actual incidence of post-meningitis hearing loss is probably underestimated because audiometric testing is only performed in clinical suspected cases. Many cases are late or never diagnosed [9]. For that reason, routine hearing evaluation is recommended in the standard follow-up program of childhood BM aiming to achieve more timely intervention [8], [14].

**Figure 1:**
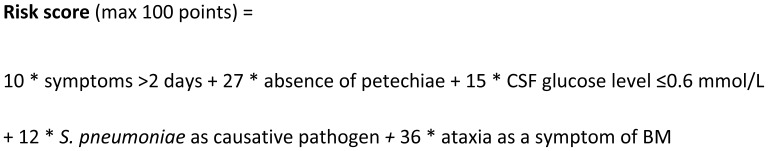
clinical prediction rule for the prediction of hearing loss after childhood BM, as presented by Koomen *et al*.

To support the recognition of patients at high risk for hearing loss after BM, Koomen *et al.* developed a clinical prediction rule (figure 1) [6]. Clinical prediction rules are (regression) models that use three or more variables from patient history, clinical course or diagnostic tests to calculate a probability of an outcome measure. These rules are potentially strong tools that are currently used in clinical decision-making [15]. The model constructed by Koomen et al. included the following independent predictors for hearing loss: duration of symptoms prior to admission, absence of petechiae, cerebral spinal fluid (CSF) glucose level, *Streptococcus pneumoniae* as causative pathogen and ataxia.

In general a prediction model does not perform well in a different cohort than the one it was constructed in. For that reason external validation in an independent cohort is essential before implementation in practice [16], [17], [18]. The aim of this study was to externally validate the existing model for hearing loss after childhood BM in a validation cohort of Dutch school-age BM survivors in order to evaluate the potential for usage in clinical practice.

## Methods

The study was approved by the medical ethics committee of the VU University Medical Center in Amsterdam. Written informed consent was obtained from the parents or guardians of all children and from the children themselves if they were at least 12 years of age.

### Development Cohort

The construction of the prediction rule was described in the original publication by Koomen *et al.* [6]. Files of the Netherlands Reference Laboratory for BM (NRLBM) were searched for children born between January 1986 and December 1994 who survived BM between January 1990 and December 1995. The NRLBM receives approximately 90% of the isolates of Dutch meningitis patients [19]. The diagnosis BM was based on the isolation of bacteria in the CSF. Exclusion criteria included a complex onset of meningitis (defined as: meningitis secondary to immunodeficiency states, CNS surgery, cranial trauma, CSF shunt infections of relapsing meningitis), pre-existent cognitive or behavioral problems, and diseases developed after BM, which could have caused cognitive or behavioral problems. These last 2 exclusion criteria were used while the cohort was also constructed for a study on academic or behavioral problems after BM [20].

Sixteen hundred and five children were eligible for inclusion and their pediatricians were approached to send the parents a letter requesting participation. Six hundred and twenty-eight children were included, and their medical records were investigated for risk factors and for perceptive hearing loss of >25 dB. Hearing loss was found in forty-three children (7%) and by reviewing medical records; information was collected on all potential risk factors for this hearing loss. Predictors univariably associated with the outcome (*p*≤0.10) were included and a prediction model was developed using multivariable logistic regression. Five risk factors for hearing loss were found to be independent determinants: duration of symptoms prior to admission longer than two days, the absence of petechiae, CSF glucose level ≤0.6 mmol/L, *S. pneumoniae* as causative pathogen and the presence of ataxia during the illness. After internal validation using bootstrapping techniques and shrinkage of regression coefficients this model was transformed into a clinical prediction rule as presented in figure 1. The scores and the matching probability of hearing loss were visually presented in a nomogram for use in clinical practice. [6].

### Validation Cohort

In 2005, the files of the NRLBM were searched again for Dutch children born between January 1993 and December 1999 who suffered from non-*Haemophilus influenzae* type b (Hib) BM between January 1997 and December 2001. The exclusion criteria were identical to those used in the original study [6]. One thousand and thirty-six children were eligible for inclusion, and the pediatricians were requested to send the parents (or guardians) an invitation letter to participate in the study. After informed consent the parents were sent screening questionnaires regarding health, learning and behavior. Three hundred and fifty eight children were included in the cohort used for validation of this model. Parallel to the approach used in the development study of Koomen *et al.* in which a prediction rule for academic and behavioral problems after BM was constructed, a nested cohort approach was used [20]. In this design only a subset of cases and controls are selected for further analysis, which decreases the necessary time and financial resources resulting in an improved efficiency [20], [21].

In the total cohort of 358 children a nested cohort of 160 children were randomly selected and invited to visit our department for academic and neuropsychological testing and for the completion of questionnaires regarding behavior and health. Forty-four of the invited children did not participate in this part of the study.

**Figure 2:**
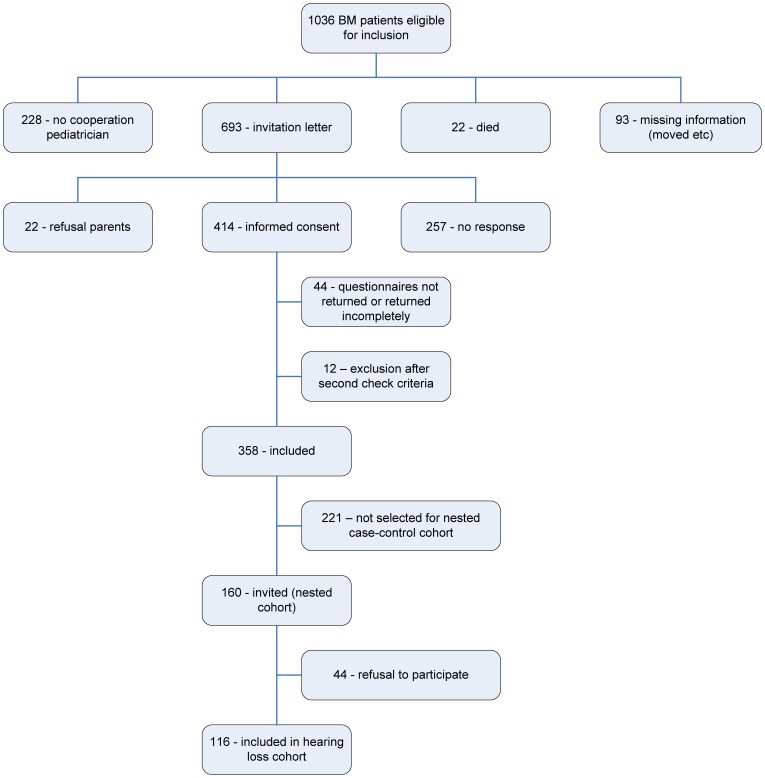
Flow chart of patient inclusion.

Again, the outcome measure “hearing loss” was defined as a unilateral or bilateral perceptive loss of >25 dB and was based on findings in these records and on parental information provided in the questionnaires about the children’s health (Dutch versions of the Child Health Questionnaire (CHQ) and the Health Utilities Index (HUI) mark 2&3) [22], [23]. Information on the risk factors for hearing loss was also collected by reviewing medical records of the pediatrician and the otolaryngologist after permission from the parents. Conductive hearing loss was not included. Finally, information about hearing loss was retrieved from 116 children. Figure 2 presents a flow chart of patient inclusion.

The risk factors evaluated for this study were the five aforementioned independent predictors included in the original model.

#### Data analysis

Univariate comparison of the distribution of patient characteristics and clinical data in the development and the validation cohort was performed by independent sample t-tests for continuous and χ^2^-tests for nominal data. Fisher’s Exact test was used if the data did not meet the criteria for a χ^2^-test. Statistical significance was considered with 2-tailed *p*-values of ≤0.05.

### Missing Data

In both the development and the validation cohort multiple imputation techniques were used for substitution of missing variables. In the development cohort a maximum of 20% of the data was missing per variable [6]. In the validation cohort no more than 5% of the data was missing per variable, resulting in 9.5% missing cases for the whole model. Imputation was repeated five times, resulting in five different datasets [24], [25], [26].

### External Validation

For external validation the discriminative ability of the model in both cohorts was compared. In all sets the model with the original regression coefficients was applied on the data of each individual child. Receiver operating characteristic (ROC) curves of all imputated datasets were constructed for these results predicting the outcome measure “hearing loss”. An average area under the curve (AUC) was calculated and compared with the average AUC of the ROC curves of the development cohort. The fit of the model in the validation cohort was tested with the Hosmer-Lemeshow test for goodness of fit. For this test the patients were grouped by decile of predicted probability and differences between expected and observed outcome in the ten groups were tested using χ^2^-tests. The five results of these χ^2^-tests of the imputated datasets were pooled [27].

The intercept in a logistic regression model results in equal averages of the predicted probabilities and the outcomes when the model is applied in the validation cohort (“calibration in the large”). The slope reflects that the regression coefficients are correctly estimated and that they yield the correct predictions in the validation cohort. We also assessed more specifically whether calibration was successful by separately testing deviations in the intercept and calibration slope when the model was applied in the validation cohort [27].

For each child in the validation cohort the individual risk score was calculated. The distribution of the number of subjects across categories according to these scores were compared between the development and the validation cohort. Positive and negative predictive value, sensitivity and specificity were calculated for different cut-off points and were compared for their clinical value with those composed with the original prediction model.

All data was analyzed using SPSS Statistics 18.0 (IBM Corporation, Somers, NY) and R (The R Project for Statistical Computing).

## Results

Table 1 presents the distribution of the patient characteristics, independent predictors and the outcome measure “hearing loss” in the development and the validation cohort. Differences in distribution of characteristics between the two cohorts were observed for the duration of symptoms before admission, consciousness, presentation of the child with meningeal irritation at admission, (duration of) dexamethasone prescription, number of children mechanically ventilated, duration of hospitalization, duration of anti-epileptic therapy and the number of children with focal neurological deficits. There were no significant differences in the distributions of the five predictors of the prediction rule. The incidence of hearing loss was 6.8% in the development cohort and 12.1% in the validation cohort.

**Table 1:** Patient characteristics of the development and validation cohort.

Characteristics	Validation cohort(n = 116)			Development cohort(n = 628)			*p*-value^f^(development vsvalidation cohort)
	No^a^			No^a^			
Age at infection (years)^b^	116	2.5	(1.8)	628	2.4	(2.0)	0.58
Male gender^c^	116	57	(49.1%)	628	356	(56.7%	0.13
***Patient history and physical examination at admission***							
Duration of symptoms prior to admission (days)^b^	113	2.4	(1.4)	621	1.9	(1.8)	0.001
**Duration of symptoms >2 days** c	113	36	(31.9%)	621	146	(23.5%)	0.059
Seizures prior to admission^c^	116	8	(6.9%)	628	57	(9.1%)	0.45
Seizures ad admission^c^	116	4	(3.4%)	628	59	(9.4%)	0.035
Decreased consciousness^c^	106	53	(50.0%)	590	401	(68.0%)	<0.001
Rectal temperature (°C)^b^	100	39.1	(1.2)	583	39.1	(1.0)	0.64
Rectal temperature ≥38°C^c^	112	94	(83.9%)	583	502	(86.1%)	0.55
Meningeal irritation^c^	109	78	(71.6%)	628	511	(81.4%)	0.018
**Petechiae** c	115	65	(56.5%)	618	336	(54.4%)	0.67
Focal neurological deficitsc, d	115	6	(5.2%)	628	58	(9.2%)	0.16
Middle ear infection^c^	93	13	(14.0%)	514	48	(9.3%)	0.17
***Laboratory tests***							
CSF leukocyte count (/uL)^b^	111	3827	(6778)	548	3736	(6719)	0.90
CSF glucose (mmol/l)^b^	110	2.2	(1.9)	543	2.2	(1.8)	0.94
**CSF glucose ≤0.6 mmol/l** c	110	33	(30.0%)	543	165	(30.4%)	0.94
CSF protein level (g/l)^b^	104	1.8	(1.5)	508	1.8	(1.7)	0.89
Causative pathogen in CSF: (total)	116			628			
* N. meningitides*		92	(79.3%)		495	(78.8%)	0.91
*** S. pneumonia***		22	(19.0%)		103	(16.4%)	0.50
* S. agalactie* (group B)		2	(1.7%)		18	(2.9%)	0.76
* E. coli*		0	(0%)		10	(2.9%)	0.38
* L. monocytogenes*		0	(0%)		2	(0.3%)	1.0
Bloodculture positive for BM causing pathogen	116	49	(42.2%)	628	300	(47.8%)	0.27
Serum leukocyte count (x10^9)^b^	116	16.2	(9.4)	613	17.8	(10.7)	0.15
***Therapy and clinical course***							
Dexamethasone prescribed^c^	110	19	(17.3%)	628	170	(27.1%)	0.029
Duration dexamethasone therapy (days)^b^	110	0.6	(1.5)	602	0.9	(1.8)	0.025
Mechanical ventilation^c^	116	14	(12.1%)	627	39	(6.2%)	0.025
Duration of hospitalization (days)^b^	115	12.9	(8.0)	618	14.7	(9.4)	0.046
Seizures during hospitalization^c^	115	8	(7.0%)	628	64	(10.2%)	0.28
Duration of anti-epileptic therapy (days)^b^	112	0.6	(2.6)	610	1.4	(5.7)	0.011
Focal neurological deficitsc, d	114	7	(6.1%)	627	94	(15.0%)	0.011
**(transient) ataxia^c.e^**	115	3	(2.6%)	628	16	(2.5%)	0.97
***Outcome measure***							
Hearing loss	116	14	(12.1%)	628	43	(6.8%)	0.052

a Number of subjects the variable was obtained.

b Mean (standard deviation).

c Number of subjects (%).

d Focal neurological deficits are defined as cranial nerve deficits, increased or decreased reflexes of arms or legs, increased or decreased tonus of arms or legs, focal convulsions and ataxia.

e (Transient) ataxia was defined as signs of ataxia, which lasted at least until discharge from the hospital, as documented in the medical records.

f P-value: independent sample t-test for continuous data; χ^2^-test for nominal data, or Fisher’s Exact test if the data does not meet the criteria for χ^2^-test.

Abbrevations: No. = number, CSF = cerebrospinal fluid.

### External Validation of the Prediction Rule

The average AUC of the ROC curves of the validation cohort was 0.78 (95% CI 0.64–0.92). In the development cohort, the reported AUC was 0.84 (95% CI 0.78–0.91) [6]. The pooled result of the Hosmer-Lemeshow test for goodness of fit of the five datasets was not significant (*p*-value 0.10), indicating good fit of the model in the validation cohort concerning the similarity of expected and observed cases of hearing loss. When the original model was applied in the validation cohort there was no significant deviation in intercept (*p* = 0.07–0.11) and calibration slope (*p* = 0.14–0.34).

### Comparison of the Distribution of Risk Score Categories

Table 2 shows the numbers of subjects across categories of the score for both the development and the validation cohort. Children without hearing loss had an average risk score of 19.6 and children with hearing 45.3. In both cohorts approximately 40% of children without hearing loss score zero points, while only 1 child of all children with hearing loss has a score of 0. While most of the children with hearing loss had a higher score, children without hearing loss were in the groups with high scores as well. Table 3 presents positive and negative predictive value, sensitivity and specificity for the different cut-off points. Using a cut-off point of zero, the sensitivity is 100% while at a score of 1–25 the negative predicted value is 97.8%. At a maximum score on the risk score the specificity is 96.1%, but decreases rapidly at a lower score. The positive predictive value is low (60%). The conclusion of these tables is that sensitivity and negative predictive value are good, but specificity and positive predictive value are poor.

**Table 2:** Distribution of children with and without hearing loss across categories of the risk score.

	Children in the cohort				Children with hearing loss				Children without hearing loss			
Risk score	Development(n = 628)		Validation(n = 116)		Development(n = 43)		Validation(n = 14)		Development(n = 585)		Validation(n = 102)	
	cumulative		cumulative		cumulative		cumulative		cumulative		cumulative	
	n (%)	n (%)	n (%)	n (%)	n (%)	n (%)	n (%)	n (%)	n (%)	n (%)	n (%)	n (%)
Score 0	236 (38)		45 (39)		0 (0)		1 (7)		236 (40)		44 (43)	
Score 1–24	86 (14)	322 (51)	14 (12)	59 (51)	2 (5)	2 (5)	2 (14)	3 (21)	84 (14)	320 (55)	12 (12)	56 (55)
Score 25–36	87 (14)	409 (65)	15 (13)	74 (64)	5 (11)	7 (16)	1 (7)	4 (29)	82 (14)	402 (69)	14 (14)	70 (69)
Score 37–63	197 (31)	606 (96)	32 (27)	106 (91)	24 (56)	31 (72)	4 (29)	8 (57)	173 (30)	575 (98)	28 (27)	98 (96)
Score ≥64	22 (3)	628 (100)	10 (9)	116 (100)	12 (28)	43 (100)	6 (43)	14 (100)	10 (2)	585 (100)	4 (4)	102 (100)

**Table 3:** Positive and negative predictive value, sensitivity and specificity for the different cut-off points of the prediction rule.

	Positive predictivevalue (%)		Negative predictivevalue (%)		Sensitivity (%)		Specificity (%)	
Risk score cut-off point	Development	Validation	Development	Validation	Development	Validation	Development	Validation
Score ≥0	6.8	12.1			100.0	100.0	0.0	0.0
Score ≥1	11.0	18.3	100.0	97.8	100.0	92.9	40.3	43.1
Score ≥25	13.4	19.3	99.4	94.9	95.3	78.6	54.7	54.9
Score ≥37	16.4	23.8	98.3	94.6	83.7	71.4	68.7	68.6
Score ≥64	54.5	60.0	94.9	92.5	27.9	42.9	98.3	96.1

## Discussion

In this study, the prediction model for hearing loss after childhood BM was validated successfully in a new independent cohort of school-age children. This is the first validated model for the identification of children at high risk for hearing loss after BM. Our recommendation is that at least the patients who are positive at one or more risk score should achieve timely and frequent hearing evaluation. It is not a replacement for standard hearing tests, but an addition to these tests, to estimate the risk for hearing loss in an early stage of disease.

Despite increasing awareness and recommendation of routine hearing evaluation in the standard follow-up of childhood BM, the amount of children whom hearing is not tested or hearing loss is even missed could be up to 25–30% [6], [14]. Relatively little is known about the clinical course of hearing loss after BM. It is not always noticeable or present directly after the infection and fluctuation and deterioration of hearing later in time might occur [28], [29], [30]. Modern follow-up protocols therefore include a prolonged period of hearing loss evaluation. The only current treatment option in complete and profound hearing loss is cochlear implantation, which may only be possible in a critical period. A good opportunity for hearing restoration could disappear within weeks since labyrinthitis ossificans makes implantation extremely difficult or even impossible [14], [31]. This prediction model can be used as a screening tool and can help to identify those children that need special attention and a long follow-up period or more frequent auditory testing even when the first test is negative.

For clinical practice the optimal cut-off point of the risk score has to be defined. Conform Koomen *et al.* we state that hearing loss should not be missed at all and therefore propose a low cut-off value of zero points [6]. In this study it was confirmed that this is the optimal cut-off point. The risk score reaches excellent sensitivity and negative predictive value at this cut-off point. Unfortunately, as one increased the sensitivity of a rule, its specificity tends to decrease (and vice versa). In clinical practice, this indicates that our prediction rule has to be used to select the children at high risk, accepting the fact that a relatively high number of children without hearing impairment will be selected as well. Although with a low cut-off point sensitivity is high, in the validation cohort one child with a score of zero points did have hearing loss, while in the construction cohort no case was missed. We conclude that at least those patients who are positive at one or more factor score should achieve timely and frequent hearing evaluation. In the lower risk group, hearing must be evaluated as well, but it may be considered to perform auditory tests less frequently of for a shorter time period. This may improve the balance between costs and benefit. It can create awareness of the importance of hearing evaluation in clinicians and the parents in an early stage of disease.

Further, BM and subsequent morbidity is considerably more prevalent in developing countries where adequate follow-up, hearing tests, financial resources and support in case of auditory deficits are scarcely available [32]. Prediction models should be developed these countries as well and may help to select and at least test the group that urgently needs to achieve hearing evaluation.

This study has several strengths. This is the first model developed and externally validated that predicts hearing loss after BM in childhood. In the last decade, many prediction models have been designed and presented in literature. Before implementation in practice is possible, external validation is an essential step [16], [17], [18]. However, the majority of proposed models have never been validated [18], [33]. Therefore, successful validation of our model is an important step forward in the development of a complete protocol for the follow-up of children who suffered from BM.

In general, the discrimination of a prediction model is interpreted to be fair, good or excellent when the AUC is or 0.7–0.8, 0.8–0.9 or 0.9–1.0, respectively. This model has an AUC of 0.84 in the development cohort and an AUC of 0.78 in the validation cohort, thus can be at least considered as fair.

Further, state of the art methodology is used and described clearly. Therefore it may be easily to reproduce in a wide range of future studies.

The validation cohort was constructed using the nested cohort approach while in the development of the rule the total initial cohort was used [6], [20]. From the 1036 patients selected from the NRLBM database, 116 were included. Most children were excluded in the first step because approached pediatricians or parents refused participation or because the parents could not be contacted due to missing or incorrect address data. From the resulting 361 children 116 were included for validation of the hearing loss model. This nested cohort design, in which only a subset of cases and controls are randomly selected for further analysis, is a known and appreciated methodology that results in more efficiency with reference to time and financial resources [21]. This stepwise construction may lead to differences in case mix between de development cohort and the validation cohort due to selection. The incidence of sensorineural hearing loss was 6.8% in the development versus 12.1% in the validation cohort (*p*-value = 0.052). This difference may be explained by the fact that in the development cohort 27% of the children were not tested for hearing loss, resulting in underestimation of the incidence [6]. Further, an increase in awareness in the period of approximately seven years between the two studies might have resulted in increased follow-up and incidence of hearing loss. Last, the selection process of the nested cohort described above may also be responsible.

Case mix differences were also found in patient characteristics and risk factors, such as: duration of symptoms >2 days, meningeal irritation, dexamethasone therapy, mechanical ventilation, duration of anti-epileptic therapy and focal neurological deficits. These differences could have been responsible for a smaller spread in the risk of hearing loss (0–91% in the development versus 2–70% in the validation cohort). This may in turn be responsible for the small drop in discrimination of the model in the validation cohort. Because the differences in case mix may partly be explained by the selection processes, we must be careful to draw conclusions from the comparison of the incidence of hearing loss and risk factors, while some selection bias may have occurred.

Though, for the external validation selection bias is not a major issue: the development cohort appeared to be a representative sample of the pediatric BM population of that time: The NRLBM receives approximately 90% of the isolates of Dutch BM patients, and the 628 children included where very similar compared with the 1605 eligible children regarding, sex, age, and causative pathogens [6].

Validation samples may differ from development samples in predictor (patient) characteristics and in outcome frequency. These differences may, as we discussed, also occur during the selection of patients in the development sample and not only depend on selection of patients in the validation sample. Differences between samples may be caused by sampling variability or true differences, and not by selection bias per se. The goal of external validation is to investigate to what extend these differences influence the generalizability of the prediction model. Sampling variability (or working with smaller validation samples) will often show a small influence on the performance of the prediction model. We showed that at low and high risk score categories, which are most important for clinical practice, the model performed equally well in the validation as in the development sample [16], [18], [34], [35].

Information bias may have occurred. Risk factors and the outcome were determined retrospectively, with a risk of bias and missing data. To minimize the chance of bias, collection and interpretation of clinical data from the medical files was performed in a standardized w ay, and consensus meetings were held when there was any doubt about how to interpret the medical files. The diagnosis “perceptive hearing loss of >25 dB” is made in the Netherlands by clinicians or audiologists by standard tests. Multiple imputation techniques were used for substitution of missing data, which is currently the most reliable strategy to deal with missing information on covariates [26], [36], [37], [38].

Further, sample size of the validation cohort was small. A frequently used general rule in the development of clinical prediction models is that the ratio events per variable (EVP) should be approximately 10 [39]. In the development cohort there were 43 events for 5 predictors included in the model, resulting in an EVP of 8.6. For external validation new insights in sample size issues are developed in recent years. It has been suggested that to detect minor changes in discrimination and calibration, and prevent type II error (the Null hypothesis of equal model performance is falsely not rejected), the validation cohort should contain at least 100 events and 100 non-events [40]. The sample size of our validation cohort is limited, and therefore small differences in calibration might have been missed.

### Future Perspectives

It is hypothesized that the distribution of BM pathogens has changed since the introduction of these vaccines and therefore the model will not perform well in the contemporary population. Since the development of the prediction rule two new vaccines against *N. meningitidis* serogroup C and high frequency serotypes of *S. pneumoniae* were introduced in the Netherlands and other western countries: Accompanied by a spontaneous decrease of *N. meningitidis* serogroup B infections this resulted in a dramatic decrease in incidence of BM. Serotype replacement with increasing incidence of infections has not yet occurred for *N. meningitidis*, but new outbreaks with non-vaccine-serotypes are possible and have occurred in history [41], [42], [43]. For *S. pneumoniae* there is a net reduction of invasive infections but an increase in infections with non-vaccine-serotypes is observed [44]. Therefore, we should be careful expecting BM to become and stay a rare disease, and prediction rules for sequelae remain valuable. In a recent study our group simulated a population without the seven serotypes of *S. pneumoniae* included in the vaccine, and showed that the model remains stable in this situation [45]. To investigate whether the long-term consequences of vaccination have impact on the performance of the model, another validation study in children that were infected more recently is planned.

Another important development is the rapidly increasing knowledge on the influence of genetics on the course of diseases. This also applies for BM and hearing loss, and in recent publications an effort was made to disclose the influence of genetic variation in the immune response and the course of the disease in children. Van Well *et al.* described a strong association between host genetic polymorphisms in pathogen recognition receptors and hearing loss after BM extracted from both development and validation cohort described in this study [46]. Additional analyses will be performed to study whether our model can be extended with genetic factors to predict post-meningitis hearing loss even more accurately.

We conclude that we created and externally validated a clinical useful tool in addition to regular auditory testing for the identification of children at high risk for hearing loss after BM. In the future larger development and validation studies must be performed, in which hearing loss is measured prospectively and genetic risk factors are included in the construction of the prediction model. In our opinion, international cooperation is the answer to the problem of decreasing incidence in the development of prediction models.
